# Learning hand in hand: Engaging in research–practice partnerships to advance developmental science

**DOI:** 10.1002/cad.20364

**Published:** 2020-09-13

**Authors:** Kelly Lynn Mulvey, Luke McGuire, Adam J. Hoffman, Adam Hartstone‐Rose, Mark Winterbottom, Frances Balkwill, Grace E. Fields, Karen Burns, Marc Drews, Melissa Chatton, Natalie Eaves, Fidelia Law, Angelina Joy, Adam Rutland

**Affiliations:** ^1^ North Carolina State University Raleigh North Carolina United States; ^2^ University of Exeter Exeter United Kingdom; ^3^ University of Cambridge Cambridge United Kingdom; ^4^ Centre of the Cell Queen Mary University of London London United Kingdom; ^5^ Riverbanks Zoo and Gardens Columbia South Carolina United States; ^6^ Virginia Aquarium & Marine Science Center Virginia Beach Virginia United States; ^7^ EdVenture Columbia South Carolina United States; ^8^ Florence Nightingale Museum London United Kingdom; ^9^ Thinktank Birmingham United Kingdom

**Keywords:** community, equality, partnership, practice, research, trust, youth

## Abstract

Developmental science research often involves research questions developed by academic teams, which are tested within community or educational settings. In this piece, we outline the importance of research–practice partnerships, which involve both research and practice‐based partners collaborating at each stage of the research process. We articulate challenges and benefits of these partnerships for developmental science research, identify relevant research frameworks that may inform these partnerships, and provide an example of an ongoing research–practice partnership.

Our assumptions about what we know and what we should investigate too often create barriers that prevent us from truly learning more. This is true in the field of developmental science, as frequently, what we “know” about development is the accumulation of evidence gathered from specific contexts with particular samples, largely reflecting WEIRD (Western, Educated, Industrialized, Rich, and Democratic) populations (Henrich, Heine, & Norenzayan, 2010). In this piece, our interdisciplinary and international team of scholars, practitioners and youth educators frame an argument for a new way forward for developmental sciences. We argue for true research–practice partnerships where academics work hand‐in‐hand with communities, including practitioners, providers, children, and adolescents, to identify critical new directions for research. These new directions should be both informed by the needs and the knowledge base of local communities and responsive to the unique assets and strengths that diverse teams bring to research–practice partnerships. We argue that research–practice partnerships in developmental science should aim to take an equity‐driven approach (Brown, Mistry, & Yip, 2019)—gaining new knowledge, as well as sharing knowledge in ways that are accessible both for advancing developmental science and applying it directly in practice (Haden, 2020). In fact, a growing body of voices in developmental science are documenting the evidence base for equity‐oriented research that directly addresses and examines inequities (Brown et al., 2019).

## 1 WHAT ARE RESEARCH–PRACTICE PARTNERSHIPS?

Research–practice partnerships involve close work bridging academic and practice‐based interests to grapple with pressing needs, grand challenges, and unanswered questions. Practice‐based fields could include community educational institutions, both formal and informal, although they may take other forms. Research–practice partnerships focused on children and adolescents are often partnerships between academic scholars (the research arm) and community educational institutions (the practice arm) with both partners seeking to explore shared questions (Bevan & Penuel, 2017). Research–practice partnerships are each unique in scope and size, but generally these partnerships aim to generate knowledge to inform practice in some form, extend beyond the life of a single research study or project, and involve equitable contribution by all partners in shaping the direction of the work, including the questions under study and the methods used to explore those questions (Penuel & Hill, 2019).

There are many benefits to a research–practice partnership, as these partnerships have the potential to improve the research itself *and* have long lasting benefits for practice. In fact, these partnerships can often foster more rigorous research than can be undertaken in other settings, for instance by testing causal impacts through experimental assignment (Haden, 2020). These rewards are substantial and numerous; however, such partnerships do involve hard work. As outlined by Farrell, Harrison, and Coburn (2019), learning about each other, negotiating roles, and building trust are essential for all steps of a research–practice partnership. Establishing equal, trusting relationships between researchers, practice‐based partners and participants is of critical importance, but requires active work, commitment and active identification of goals, agendas and assumptions that each member of the team brings to the partnership. Recently, the William T. Grant Foundation put forward a framework for evaluating research–practice partnerships, noting the importance of five dimensions for successful research–practice partnerships: (1) building trust between partners, (2) ensuring that the research is rigorous, (3) supporting the practice‐based organization, (4) generating knowledge that can inform practice, and (5) building capacity for the practice‐based organization's success (Henrick, Cobb, Penuel, Jackson, & Clark, 2017). This framework establishes benchmarks for measuring effectiveness, but can also inform the development and sustainability of such partnerships.

We argue that these partnerships are essential for the future of developmental science as a field. We task researchers to look those in their community as they first consider research topics—with the goal of partnering with practice‐based communities at every stage, from developing research questions, implementing projects and learning from the findings. The practice‐based institutions should not merely be viewed as sites where subjects can be sampled (Haden, 2020), but as places where partners can be found to answer mutually beneficial questions. Moreover, we argue that children and adolescents, themselves, can, and perhaps should, be part of the research–practice partnership, informing the research design, aiding in interpreting and disseminating the findings, and shaping new directions for both policy and practice. Educators have increasingly recognized cultural funds of knowledge that their students bring to the classroom (González, Moll, & Amanti, 2006). Further, there are burgeoning attempts to bring youth into the research process at each stage of the research (Foster‐Fishman, Law, Lichty, & Aoun, 2010). Aligned with these attempts to value youth voices, we argue that potential participants (children, adolescents, young adults, and families) possess important perspectives, key knowledge and insights that we may be missing if we do not also bring their voices to our research–practice partnerships. Thus, we suggest that an equitable partnership needs to include not only the practitioners that work with youth, but also to focus on creating spaces and opportunities for the young people whose development we are interested in understanding to contribute to the research agenda.

This can be a challenge for researchers, who are used to articulating research questions upfront and systematically testing their hypotheses. As noted by Denner, Bean, Campe, Martinez, and Torres (2019), research–practice partnerships can be uncomfortable and require a willingness for the partners to critically assess roles that hierarchies and culture play in creating opportunities for establishing and aligning priorities, breaking down stereotypes and working collaboratively. In our own work, we have found that allowing team members to really get to know each other and fostering regular communication, including as many opportunities as possible for in‐person communication, are essential for partnerships that build trust. Depending on the research–practice partnership, this may take different forms. For instance, regular meetings at each other's spaces (i.e., the research campus and/or the site) may help. Or informal visits to the practice‐based sites, not during formal research process, may aid in building trust.

## 2 FRAMEWORKS TO SUPPORT RESEARCH–PRACTICE PARTNERSHIPS

Developmental science as a field has long recognized the importance of context and culture in research. As outlined by Fisher et al. (2002, 2016), work with youth, especially marginalized youth, should always include attention to the cultural and contextual factors that are relevant for the populations of interest in all steps of the research, and should seek and value community and participant perspectives during the research process. Our perspective on research–practice partnerships draws on these best practices for ethical research and aligns well with research approaches that recognize the importance of context.

One such framework is community‐based participatory research (CBPR), which refers to research approaches that directly engage community members in identifying and developing as well as in evaluating strategies and approaches to solve problems (Mikesell, Bromley, & Khodyakov, 2013). While CBPR has often focused on health‐oriented problems, as evidenced by the focus on CBPR in funding agencies such as PCORI (Patient‐Centered Outcomes Research Institutes), this model can be applied to many questions relevant for developmental scientists as well. Namely, CBPR pushes for ensuring that research is conducted in an ethical manner, by recognizing the Belmont Principles of autonomy, beneficence and justice in CBPR studies, with the focus of the work centered on the interests of the community (Mikesell et al., 2013). While CBPR models inherently value the voices of the participants in the research process (this is quite rare in developmental research), findings reviewing CBPR with youth find that only 15% of the studies included youth directly in some phase of the research process (Jacquez, Vaughn, & Wagner, 2013). This is both a missed opportunity and ethically questionable. Developmental science as a field should move towards not only seeking out, but also valuing, the voices of the youth we are studying (see Foster‐Fishman et al., 2010, for one example of how youth can be participatory members of a qualitative research program). Youth can provide a wealth of benefits to the improvement of a study. Namely, youth can contribute novel insight into key questions that we might study, offer suggestions and strategies as to how to best collect data within their communities, and can also provide innovative ideas as we seek to analyze, interpret, and disseminate our findings.

**FIGURE 1:**
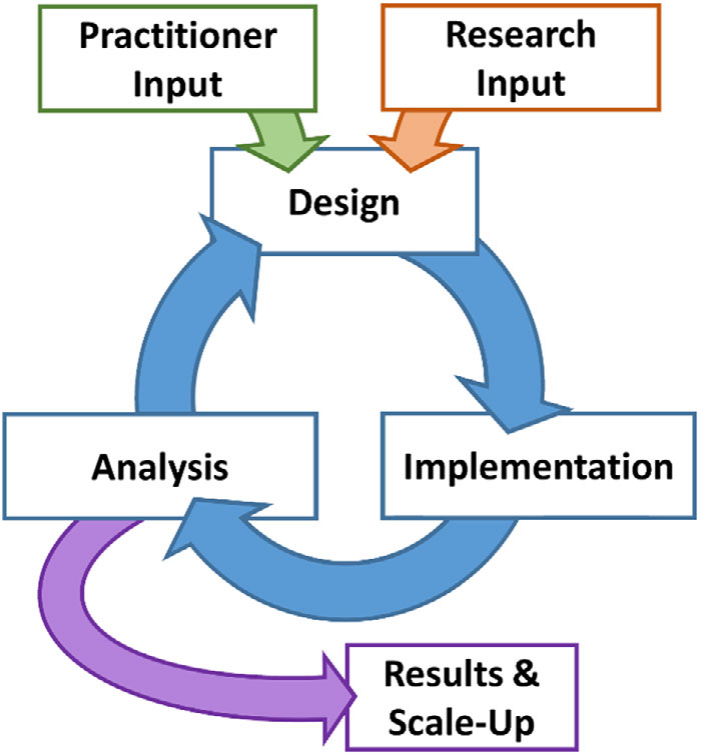
Design‐based research process

An additional set of guidelines that may be an especially helpful guide when seeking to begin research–practice partnerships is design‐based research principles (Barab, 2014). Design‐based research was developed as an method that involves bridging the gap between research and practice, allowing for educators to design and test novel educational interventions with attention to the importance of refining the approach following analysis of multiple sources of data (Anderson & Shattuck, 2012). Design‐based research recognizes the important contributions of theory as well as data derived from the specific educational context to inform development, design, and re‐design of the intervention (Barab, 2014). Design‐based research also allows for the development of more generalizable theoretical ideas, which can be tested in diverse settings. As illustrated in Figure 1, design‐based research is an ongoing process and does not involve simply identifying a research question, testing research questions or an intervention, and reporting the results. Rather, design‐based research involves shapring the research goals with input from both researchers and practitioners, implementing the research or intervention collaboratively, working together to analyze the findings and finally drawing conclusions and examining ways in which the findings can be applied more broadly or the intervention can be scaled‐up for use in other settings. Furthermore, design‐based research is founded upon collaboration and partnership between researchers and practitioners. While this model was originally developed to bring educators into the research process, frontlining their experiences and knowledge as part of the research process, the model of design‐based research can be applied to any research–practice partnership. For developmental scientists, building on design‐based research principles may mean iteratively testing and refining interventions, but even outside of this approach, research can draw on these principles by valuing the voices and perspectives of the community partners and the researchers at all stages of the research.

Importantly, these are not new approaches. In fact, action research models, which aim to address problems through cycles of planning, taking action and seeking knowledge to inform next steps, were first developed in order to foster social change and transformation in the 1940s (Lewin, 1946). Action research is still a key approach, particularly in educational settings, for seeking evidence to inform new practices and refined policies (Stringer, 2013). Recent research using action research approaches has involved partnership between teachers, researchers and facilitators, noting the importance of defining roles and maintaining communication to ensure success of such projects (Platteel, Hulshof, Ponte, van Driel, & Verloop, 2010). Each of these frameworks can inform research–practice partnerships that have a likelihood of advancing developmental science and meaningfully transform the practice of youth programming.

## 3 A CASE STUDY: STEM TEENS

We now provide an overview of our current, on‐going research–practice partnership as an example of how such partnerships can develop, function, evolve, and contribute to the field of developmental science. The STEM Teens project is a collaboration between researchers and practice‐based educational partners at six informal science learning sites (ISLS) in the United States and the United Kingdom, which all have formal, extracurricular programs for adolescents, called youth educator programs. These programs vary in terms of the age and number of adolescents they serve, how the teens are trained and recruited, exactly what the teens do as part of the programs and whether and how the teens are compensated for their participation. However, at each site the teens both (1) learn STEM content in some form and (2) engage in interfacing with the public as educators. The sites are all diverse in terms of their missions and the populations they serve. The sites include Centre of the Cell, a biomedical science learning center in London, UK; EdVenture, a children's museum in Columbia, South Carolina; The Florence Nightingale Museum, a medical history museum in London, UK; Riverbanks Zoo & Garden, a large zoological park and garden in Columbia, South Carolina; Thinktank, a science museum in Birmingham, UK; and the Virginia Aquarium & Marine Science Center, an aquarium in Virginia Beach, Virginia. Our academic team is equally diverse, with five universities represented and scholars from diverse fields including developmental psychology, science education, cancer biology, and biological sciences. Finally, we also have an external program evaluator, who provides a process‐based evaluation of our partnership on an ongoing basis.

This partnership is a Science Learning + project—one of five projects jointly funded by the National Science Foundation in the United States and the Wellcome Trust and Economic and Social Research Council in the United Kingdom aimed at examining informal science learning. When the grant call was released for the Science Learning + projects, some of the academic team members began discussing possible research ideas. Quickly, the project began to take shape. One of the strengths of our collaboration as it developed was that two of our team members held positions both in informal learning sites and in academic institutions, helping to bridge gaps in language, terminology, and objectives. Further, as the project developed, the team grew to include both “boots on the ground” practitioners at the informal learning sites (primarily members of the education teams who facilitate the youth educator programs) as well as administrators at the sites. This allowed the team to consider multiple perspectives as we defined and developed our research questions collaboratively.

Initially, the project had three aims:

1. **Outcomes for teens**—To measure the longitudinal impact of participation in an extended youth educator experience in an informal science learning sites on: (a) educational aspirations and trajectories (Boxer, Goldstein, DeLorenzo, Savoy, & Mercado, 2011); (b) STEM self‐efficacy (Bandura, Barbaranelli, Vittorio Caprara, & Pastorelli, 2001); (c) attitudes and beliefs regarding participation in the STEM fields (Eccles & Wang, 2016; Liben & Bigler, 2002), including both stereotype acceptance and attitudes regarding inclusivity of STEM environments (Mulvey, Hitti, & Killen, 2010); (d) ability, interest, and engagement in STEM (Eccles & Wang, 2016; Wang & Degol, 2013); (e) personal occupational values and occupational values associated with STEM fields (Eccles & Wang, 2016; Weisgram, Bigler, & Liben, 2010; Weisgram, Dinella, & Fulcher, 2011); and (f) science/math identity (Aschbacher, Li, & Roth, 2010).
2. **Outcomes for visitors**—To compare visitor engagement with, and learning from, exhibits in informal educational centers when they interact with a youth educator, relative to outcomes of interacting with an adult educator or no educators present, with a focus on: (a) interest in exhibit topic and in science in general (Waller, Peirce, Mitchell, & Micheletta, 2012); (b) time spent at exhibit; (c) attitudes towards inclusivity of ISLS (Dawson, 2014); and (d) effect of conversation content (e.g., gendered conversation styles) on depth of recall of science knowledge and conceptual understanding related to the exhibit topic (Leman, Skipper, Watling, & Rutland, 2016).
3. **Outcomes across demographics and STEM sites**—To examine differences in visitor engagement based on participant characteristics such as socio‐economic status, age, gender, ethnicity and stereotype knowledge with a focus on: (a) interest in exhibit topic and in science in general (Waller et al., 2012); (b) time spent at exhibit; (c) attitudes towards inclusivity of ISLS (Dawson, 2014); and (d) depth of recall of science knowledge related to the exhibit topic and to compare outcomes of extended youth educator experiences across different types of ISLS, including zoos, aquariums, and museums.

Now, 3 years into the project, these are still our core aims and we are exploring each with care. Crucially, what we have learned as a result of our partnership is that listening to the voices of the youth educators and the visitors opened a wealth of new questions, new approaches, and new possibilities. Thus, the partnership has evolved even beyond our initial research–practitioner partnership as we have learned from our youth educators.

## 4 LEARNING TOGETHER

As an example, over the past year, we have sought more and more opportunities to hear the voices of adolescents themselves and to bring their perspectives into the project. This has developed in a few different ways. First, each year, the researchers and practitioners meet in person as a team, rotating between visits in the U.S. and the UK sites. As part of these meetings, we invite some of the teens to join us to talk, share, and learn together. For instance, in our first year, we met with a panel of teens at EdVenture to get their perspectives on what we were learning, share with them some of our findings, and hear their thoughts. Additionally, in 2019, on two occasions, teens from EdVenture had the opportunity to visit the museum partners in London, sharing their observations with the project team. This year, we have formalized this even more: one of the alumni from the program at Thinktank conducted qualitative interviews with current youth educators at Thinktank to gather deeper insight into their experiences with the program. These interviews provided exciting insight that we had not captured with quantitative data. Most importantly, the alumnus of the Thinktank program was able to garner insights from the teen interviewees by drawing on their own experiences that would not have been available to a member of the academic research team. Further, as these interviews were so insightful, we are now expanding this project and interviewing teens at several sites, extending our initial project aims. These conversations can help inform how we interpret the findings from our survey‐based research. One of our early surveys with visitors to our sites documented that gender stereotypes about STEM declined with age from early childhood to adolescence, and that these stereotypes were not dependent on whether the child interacted with an educator at the site or not (McGuire et al., 2020). Our educators in these sites do want to support counter‐stereotypes about who can and should be a scientist and actively seek opportunities to provide counter‐stereotypic examples.

As another example, we are learning that visitors have different experiences depending on who they interact with at the learning sites. Specifically, visitors reported greater interest after interacting with a youth educator than just the exhibit, and perceived that they learned more if they interact with an educator (youth or adult) rather than just the exhibit itself (Mulvey et al., 2020). Participants in middle childhood recalled more information from the exhibit when they engaged with a youth educator. Adult visitors reported greater interest after an interaction with a youth educator than with the exhibit alone or an adult educator. They also perceived that they learned more if they interacted with an educator (youth or adult) than just by visiting the exhibit. They also perceived that they learned more if they interacted with a youth educator rather than an adult educator (Mulvey et al., 2020). After sharing these findings with the teens themselves, we encouraged them to take ownership of a research project to further understand why youth educators appear, at times, to be more effective than other education opportunities at our sites. This process informed follow‐up questions that are currently being used in a new survey of the learning site visitors. Further, over the past year the teens at a few of our sites have joined us as researchers. They have been collecting data on visitor engagement, tracking how long visitors stay at an exhibit, and recording key information about the composition of the visitor family and the exhibit itself. Data collection for this project is still ongoing, however we are excited about what we can learn, not only about the youth educators, but with them as equal partners in the investigation.

Research–practice centered approaches, which involves young people as partners is not a wholly novel concept: carefully constructed guidelines are available from other disciplines such as health and social care (Kirby, 2004). They present useful principles and important questions to consider when planning for meaningful research–practice partnerships. Developmental scientists can adopt these guidelines when implementing research–practice partnerships strategies.

## 5 PARTNERING FOR DISSEMINATION

We have also committed to equitable partnership for dissemination of our findings. We believe that research–practice partnerships need to include dissemination plans that involve sharing information in a variety of ways and to a range of stakeholders. In addition to dissemination to academic journals, we prioritize dissemination to practice‐based communities and to the participants and their families themselves. As a few examples, we fully involve all members of the team in every scientific publication, encouraging all team members to play a role in manuscript preparation. Further, we present the findings as partners, with academics and practice‐based partners presenting side‐by‐side at both academic and practice‐based conferences (Deere, Fields, Rutland, McGuire, & Iqbal, 2019). Additionally, we seek opportunities to share what we have learned with practitioners and educators at other informal learning sites. Both research and practice‐partners led a workshop together at an American Zoological Association (AZA) regional meeting at the Chattanooga Zoo for current informal science educators about best practices for developing and studying youth educator programs. We foresee the involvement of youth authors on upcoming publications disseminated in a space that is meaningful and accessible to them, such as online or in youth‐focused magazines. Thus, we aim to approach our dissemination with a broad and equity‐centered lens in mind, always seeking to consider what audiences would benefit from our findings and how to ensure that our work gets to those audiences in ways that are accessible and meaningful to those audiences.

Dissemination activities that align with this new model for developmental science might take a multitude of formats. First, we strongly support aims to disseminate academic findings in open‐access journals, if at all possible. Often practitioners working with youth do not have access to research findings because of the high cost of subscribing to databases where research articles are hosted. Research is increasingly documenting the benefits of disseminating findings through open‐access outlets (Eysenbach, 2006; Swan, 2007). Planning for these costs early in a project, for instance, by advocating to funders for the importance of open access publication costs, may help to ensure that findings can be shared in open‐access formats. In our current project, our funders have been gracious in providing support for open‐access publishing. Additionally, findings could be disseminated in multiple formats. For example, while an academic publication may present the formal results of a study, the same findings can be shared through workshops with practitioners, newsletters to relevant stakeholders, and in publications and magazines through professional organizations. In fact, these dissemination efforts can lead to new insights. Our team led a workshop with zoo educators at a regional meeting of the American Zoological Society (Burns et al., 2020). The diverse perspectives of the zoo educator participants led to a rich discussion that is shaping our next questions. Further, the participants are taking what they learned directly back to their sites to help refine their own programs. Our team also led a workshop at the ESCITE conference for academics and practitioners (Deere et al., 2019) that allowed us to reach out to an international audience of practitioners. Thus, dissemination can take many forms, but we advocate for ensuring that findings are shared outside of academic circles quickly, and in formats that are of immediate use to organizations, educators and practitioners working with youth.

## 6 A CALL TO ACTION

What we have learned and are still learning over the course of this project is that, while challenging, research–practice partnerships have the potential to forward the field of developmental science in exciting new and novel ways. These partnerships provide the opportunity for the emergence of new questions, new observations, and new voices. Further, they allow us to notice, observe and analyze factors, contexts, and situations that shape development in surprising and unexpected ways. Given the riches that these partnerships offer to the science of human development, we call developmental scientists to action—reach out, seek out, engage, and work hand‐in‐hand with the practice‐based communities serving youth.
